# Disentangling the effects of *terroir*, season, and vintage on the grapevine fungal pathobiome

**DOI:** 10.3389/fmicb.2023.1322559

**Published:** 2024-01-17

**Authors:** Carla Mota Leal, Adrienn Geiger, Anna Molnár, Kálmán Z. Váczy, Glodia Kgobe, Zsolt Zsófi, József Geml

**Affiliations:** ^1^ELKH-EKKE Lendulet Environmental Microbiome Research Group, Eszterházy Károly Catholic University, Eger, Hungary; ^2^Doctoral School of Environmental Sciences, Hungarian University of Agricultural and Life Sciences, Gödöllő, Hungary; ^3^Food and Wine Research Institute, Eszterházy Károly Catholic University, Eger, Hungary; ^4^Institute for Viticulture and Enology, Eszterházy Károly Catholic University, Eger, Hungary

**Keywords:** DNA metabarcoding, fungi, grapevine trunk diseases, microbiome, plant pathogen

## Abstract

The composition, diversity and dynamics of microbial communities associated with grapevines may be influenced by various environmental factors, including *terroir*, vintage, and season. Among these factors, *terroir* stands out as a unique possible determinant of the pathobiome, the community of plant-associated pathogens. This study employed high-throughput molecular techniques, including metabarcoding and network analysis, to investigate the compositional dynamics of grapevine fungal pathobiome across three microhabitats (soil, woody tissue, and bark) using the Furmint cultivar. Samples were collected during late winter and late summer in 2020 and 2021, across three distinct *terroirs* in Hungary’s Tokaj wine region. Of the 123 plant pathogenic genera found, *Diplodia, Phaeomoniella*, and *Fusarium* displayed the highest richness in bark, wood, and soil, respectively. Both richness and abundance exhibited significant disparities across microhabitats, with plant pathogenic fungi known to cause grapevine trunk diseases (GTDs) demonstrating highest richness and abundance in wood and bark samples, and non-GTD pathogens prevailed soil. Abundance and richness, however, followed distinct patterns *Terroir* accounted for a substantial portion of the variance in fungal community composition, ranging from 14.46 to 24.67%. Season and vintage also contributed to the variation, explaining 1.84 to 2.98% and 3.67 to 6.39% of the variance, respectively. Notably, significant compositional differences in fungi between healthy and diseased grapevines were only identified in wood and bark samples. Cooccurrence networks analysis, using both unweighted and weighted metrics, revealed intricate relationships among pathogenic fungal genera. This involved mostly positive associations, potentially suggesting synergism, and a few negative relationships, potentially suggesting antagonistic interactions. In essence, the observed differences among *terroirs* may stem from environmental filtering due to varied edaphic and mesoclimatic conditions. Temporal weather and vine management practices could explain seasonal and vintage fungal dynamics. This study provides insights into the compositional dynamics of grapevine fungal pathobiome across different microhabitats, *terroirs*, seasons, and health statuses. The findings emphasize the importance of considering network-based approaches in studying microbial communities and have implications for developing improved viticultural plant health strategies.

## Introduction

The complex interplay between the grapevines and their environment extends beyond the mere physicochemical properties of the soil, it highly possibly resonates within shaping the composition, diversity, and dynamics of the microbial communities associated with grapevines, which play a crucial role in grapevine health and quality (Gladstones, 1992; Van Leeuwen and Seguin, 2006; OIV, 2010; Vaudour et al., 2015; Bokulich et al., 2016; Belda et al., 2017; Hunter et al., 2020; Pauvert et al., 2020; Gobbi et al., 2022). Among these environmental factors, *terroir*, vintage, and season stand out as assumed key determinants of the pathobiome composition and dynamics (Bokulich et al., 2014; Pauvert et al., 2020; Bekris et al., 2021).

*Terroir*, encompassing the unique combination of soil, climate, and topography of a vineyard, has a profound influence on the physiology of grapevines, potentially influencing the composition and interactions of the pathobiome within grapevine microhabitats (Hunter et al., 2020; Gobbi et al., 2022). However, the specific mechanisms by which *terroir* shapes the pathobiome remain largely unexplored. Vintage, the year in which grapes are harvested, is another environmental factor known to influence grapevine characteristics, potentially through its impact on the pathobiome (Bekris et al., 2021). By examining the microbial communities at different stages of the growing season, we can gain insights into the dynamics of the pathobiome and its potential influence on quality. Furthermore, season, the specific time of year during which grapes are grown and harvested, can further modulate the composition of the grapevine microbiome. The unique environmental conditions of each season, such as temperature and rainfall, can influence the abundance and diversity of microbial taxa within grapevine microhabitats (Pauvert et al., 2020).

The application of network-based approaches, particularly unweighted and weighted metric analysis, provides an avenue to discern the structure and complexity of these microbial communities. By quantifying the interactions among microbial taxa and assessing their relative importance within the network, this approach helps to elucidate the intricate interplay between microbial taxa and their environment (Herren and McMahon, 2017; Banerjee et al., 2018; Yousefi et al., 2023). Unweighted network analysis, based on presence/absence of taxa, provides insight into community structures and interactions that may be driven by environmental filters or competitive exclusion. In contrast, weighted networks, accounting for the abundance of different taxa, enable detection of more subtle and potentially important interactions that are often overlooked in unweighted analysis, including those influenced by the relative abundance of taxa (Barberán et al., 2012; Yousefi et al., 2023). This methodology allows for a deeper understanding of community structures and interactions that may be driven by environmental filters or competitive exclusion (Barberán et al., 2012; Banerjee et al., 2018; Yousefi et al., 2023).

Amidst the vast vineyards and microbial communities, the pathobiome plays a critical role in plant health by maintaining a balanced ecosystem that limits the growth and spread of pathogens (Vayssier-Taussat et al., 2014; Brader et al., 2017; Bass et al., 2019; Pauvert et al., 2020). Understanding the composition and dynamics of the pathobiome is essential for developing effective strategies to manage grapevine diseases and improve vineyard sustainability. Inside the grapevine’s pathobiome, the specter of Grapevine Trunk Diseases (GTDs) poses a significant threat. GTDs, often attributed to wood endophytes with the potential to switch to a pathogenic lifestyle, present a significant threat to the current vineyard sustainability (Sakalidis et al., 2011; Laurent et al., 2020; Bettenfeld et al., 2020). The prevalence of these diseases, and currently considered the most destructive biotic factor of grapevines worldwide, exacerbated by factors such as vineyard intensification and environmental changes, makes them a focal point in the examination of the grapevine pathobiome (Songy et al., 2019; Bettenfeld et al., 2020).

Despite previous studies on microbiome *terroir* and seasonality in grapevines (Bokulich et al., 2014; Gilbert et al., 2014; Knight et al., 2015; Zarraonaindia et al., 2015; Gobbi et al., 2020, 2022; Geiger et al., 2022), our understanding of the extent to which microbial communities differ among *terroirs* and the significance of these differences at varying spatial scales is still limited (Gobbi et al., 2022). Furthermore, there is increasing evidence indicating compositional differences among microbiomes of different grapevine parts or microhabitats. This suggests the role of strong niche-based processes in community assembly (Singh et al., 2018; Del Frari et al., 2019; Martínez-Diz et al., 2019; Swift et al., 2021; Geiger et al., 2022; Molnár et al., 2022). Yet, it remains unknown whether the effect of environmental differences on microbiome communities varies among these microhabitats. For example, it is still unclear whether soil or bark microbial communities, which are more exposed to the elements, exhibit greater differences among *terroirs* than microbial communities in living inner woody tissues, such as the xylem and phloem.

To address these knowledge gaps, our study embarks on an exploration of the composition, diversity, and dynamics of grapevine plant pathogenic fungi. We employed high-throughput molecular techniques, including metabarcoding and network analysis, to characterize the microbial communities associated with different grapevine microhabitats, and its possible influences by *terroirs*, vintage, seasons, and health status. Specifically, we are looking at grapevines that are symptomatic vs. asymptomatic for Esca-type grapevine trunk disease, all of the same cultivar (Furmint) and rootstock. Previous studies have investigated the microbiome of grapevines, but our study is the first to systematically compare the pathobiome composition and dynamics across different *terroirs*, seasons, and health statuses. Accordingly, we have formulated the following hypotheses: (1) The grapevine fungal pathobiome differs among different microhabitats, with distinct microbial communities inhabiting soil, bark, and wood tissue; (2) The diversity, abundance, and distribution of the pathobiome are influenced by different parameters, such as *terroir*, seasonality, vintage aspects, and plant health status. The findings from this study are expected to provide a more understanding of environmental factors that influence the diversity and distribution of pathogenic, offering valuable insights for better understanding of the pathobiome in viticulture. By employing network analysis, this study provides a more in-depth understanding of the structure and complexity of these microbial communities, which helped in uncovering microbial interactions and dynamics within these ecosystems.

These findings have significant implications for viticulture, as they provide a deeper understanding of the factors that influence the composition and dynamics of the grapevine pathobiome. This knowledge can be used to develop targeted interventions to control Grapevine Trunk Diseases and improve overall vineyard sustainability. Moreover, our results have broader implications for microbial ecology, offering a novel perspective on the influence of environmental factors on microbial communities.

## Materials and methods

### Study area and sampling

Samples were collected in three vineyards in the southern Tokaj wine region, Hungary: in the Szt. Tamás dűlő near Mád (48°11ʹ29.9”N 21°17ʹ39.0ʹE), and the southern (48°06ʹ27.1”N 21°21ʹ59.2ʹE) and northern (48°08ʹ46.3”N 21°22ʹ37.2ʹE) slopes of Nagy-Kopasz Hill near the town of Tokaj. These *terroirs* represent different mesoclimatic and edaphic conditions. In each vineyard, we selected eight grapevines; four of which had been marked as symptomatic, exhibiting typical leaf symptoms of the Esca type of Grapevine Trunk Disease (GTD), and four symptomless neighboring plants selected for each symptomatic grapevine. Samples were collected from the same marked plants at four sampling times: in February and August of 2020 and 2021.

All sampled grapevine plants were of the cultivar *Furmint*, an old and popular white variety endemic to the Carpathian Basin. This is the principal cultivar of the Tokaj wine region used to produce the botrytized *Aszú* wines, as well as dry white wines. The vineyards, aged between 26 to 44 years, featured the same scion (*V. vinifera* cv. *Furmint* clone T85) grafted on the same rootstock (*V. berlandieri* x *riparia* Teleki Kober 5BB). All were managed identically, with cordon training and the same conventional plant protection regime.

Three different microhabitats were sampled for each plant: bark tissue, perennial woody tissue, and bulk soil. The soil samples were taken from four sampling points within 50 cm around the trunk of the selected grapevine, to a depth of 20 cm. The samples were combined into a composite sample for each of the four grapevines per *terroir* and microhabitat. The bark was peeled with sterile scalpel at four points of the trunk, while woody tissue was sampled from underneath the four peeled areas of the trunk with a drill that was sterilized with a 4% (v/v) bleach solution between plants.

The samples were kept in a cooler in the field and, within five hours of collecting, were transported to the laboratory and stored at −80°C. A total of 288 composite samples were collected, representing the three microhabitats in four symptomatic and four asymptomatic plants per vineyard, sampled over two seasons across two years. All samples were lyophilized for a minimum of 72 h and homogenized using a tissue lyser and steel beads prior to DNA extraction.

### Molecular work

Metagenomic DNA was extracted from approximately 0.5 mL of lyophilized and homogenized soil, bark, and woody tissues from each sample using the NucleoSpin® Plant and Soil DNA isolation kit (Macherey-Nagel Gmbh & Co., Düren, Germany). This extraction was performed according to the manufacturer’s protocol for both the tissue and soil samples. The extracted DNA samples were PCR amplified using primers fITS7 (Ihrmark et al., 2012) and ITS4 (White et al., 1990). The PCR conditions were as follows: one cycle of 95°C for 5 min; then 35 cycles of 95°C for 20 s, 56°C for 30 s, and 72°C for 1.5 min; and concluded with one cycle at 72°C for 7 min. Prior to PCR, the DNA samples were normalized for concentration.

To link the sequences to the sample source, a second PCR was performed using the same primers, which this time were equipped with Illumina adaptors and sample-specific, 8-nucleotide barcodes. The normalization of DNA extracts, PCR reactions, and 250-base paired-end Illumina NovaSeq sequencing of amplicon libraries were conducted at BaseClear B.V. (Leiden, the Netherlands) and at BIOMI Kft. (Gödöllő, Hungary).

Besides DNA metabarcoding, the inner wood samples have also been used to isolate fungi on agar media to obtain more information on the pathobiome related to GTDs. The entire ITS rDNA region of *ca.* 800 cultures has been sequenced and have also been detected by metabarcoding (Geiger et al., unpublished data).

### Bioinformatic work

Raw DNA sequences were processed with the *dada2* package (Callahan et al., 2016), which is implemented in R (R Core Team, 2020). This package is designed to resolve fine-scale DNA sequence variation and offers improved elimination of artifactual sequences. Since *dada2* does not involve clustering sequences into Operational Taxonomic Units (OTUs), and is robust in removing spurious data, the output of unique Amplicon Sequence Variants (ASVs) captures both intra- and interspecific genetic variation of fungi found in the samples. This allows for the exploration of strain-level differences in inter- and intraspecific interactions.

Based on the quality score profiles, all forward and reverse reads were truncated to 250 and 200 bp, respectively, and were quality filtered, with the maximum number of expected errors (maxEE) allowed in a read set to 2. The filtered reads were denoised, the two-directional reads were merged, and clustered into sequence variants, which were later subjected to chimera filtering.

Taxonomic assignments of fungi were made based on the UNITE database of reference sequences, which represent all fungal Species Hypotheses (SHs) based on a dynamic delimitation (Kõljalg et al., 2013), using USEARCH v. 11 (Edgar, 2010). We assigned ASVs identified to at least the genus level to putative functional groups using the curated reference database of FungiTraits ver. 1.2 (Põlme et al., 2020).

In this paper, we investigate plant pathogenic fungi within the main genera, highlighting those known to be associated with GTDs, according to our former literature search (Geiger et al., 2022). All sequences of fungal ASVs analyzed in this paper have been deposited in GenBank (OQ370580-OQ371285).

### Statistical analyses

All statistical analyses were performed in the R environment for statistical computing (R Core Team, 2020). The ASV table was normalized for subsequent statistical analyses by rarefying the number of high-quality fungal sequences through random subsampling of the smallest library (15,246 sequences) using the *rrarefy* function in the *vegan* R package (Oksanen et al., 2013). The resulting matrix contained 1,616,808 read counts of 5,858 fungal ASVs that served as input for the subsequent analyses.

Rarefied fungal read abundance and ASV richness were compared among samples according to categorical variables, i.e., microhabitat (bark, living wood, and bulk soil), *terroir* (North, South and Szt. Tamás), vintage (2020 and 2021), season (late winter and late summer) and health state (symptomatic and asymptomatic), using analysis of variance (ANOVA) and Tukey’s HSD test in R. ASV richness and rarefied read abundance of plant pathogens were graphically presented as boxplots using the *ggplot2* R package (Wickham, 2016).

To visualize differences in fungal community composition among the samples, we used the *vegan* R package (Oksanen et al., 2013) to run non-metric multidimensional scaling (NMDS) with Bray-Curtis’s distance measure on the Hellinger-transformed ASV table, using the *metaMDS* function in *vegan* with 999 permutations in R, and a secondary matrix containing variables such as sampling source, vintage, season, health, and *terroir*. In addition, we performed PerMANOVA (*adonis*) using *vegan* to estimate the amount of variation explained by the variables, which were tested for significance independently and by including significant variables in a combined model that accounted for any correlations among them.

Climatic variables were measured using on-site weather stations at multiple *terroirs* in the Tokaj wine region. Temperature and precipitation changes during the two vintages (2020 and 2021) and seasons (Late Winter and Late Summer) were assessed via the stored climatic data at met.boreas2.hu/eke/ (Local Hungarian Meteorological Service, 2023). The data were subjected to permutational multivariate analysis of variance using the *adonis* function in the vegan R package (Oksanen et al., 2013). The resulting table (Table 1) illustrates the proportion of variation (%) in fungal community composition explained by different climatic variables in our seasons and vintages.

**Table 1:** Proportion of variation (%) of the temperature and precipitation recorded of the area of the fungal community being explained by different seasonality (Late winter and Late summer), and year/vintage (2020 and 2021) calculated with permutational multivariate analysis of variance, based on the fungal community matrix.

	Temperature		Precipitation	
	%	*p* value	%	*p* value
Late winter	1.188	**0.0012**	0.239	0.7607
Later summer	1.179	**0.0011**	1.074	**0.0031**
2020	1.09	**0.0021**	2.215	**0.0001**
2021	0.646	**0.0451**	0.781	**0.035**

Followed by significant *p* values from variance analysis. Significant results are in bold.

We inferred and visualized possible interactions among ASVs of plant pathogenic fungi in all the three different sources as a cooccurrence table and network graph, using the *cooccur* and *visNetwork* packages, respectively (Veech, 2013; Griffith et al., 2016; Almende et al., 2019) based on presence-absence matrices. The *cooccur* package was used to select pairs of ASVs that co-occurred significantly more often or less often than expected by chance. We then used the co-occurrence table for network visualization, indicating both positive and negative pairwise correlations.

The weighted and unweighted network analysis was performed using the R package *igraph* (version 0.10.1, Csardi and Nepusz, 2006). It was performed descriptive and comparative statistics on network measures, where they were statistically analyzed to assess the effects of microhabitat, *terroir*, season, vintage, and health status. The statistical metrics analyzed: Average degree, network density and modularity (unweighted network analysis), and degree and betweenness centrality (weighted network analysis). See material and methods section of network metrics analysis for further details.

Furthermore, we performed indicator species analysis using the *multipatt* function in the *indicspecies* package (De Cáceres et al., 2012) with 999 permutations, to identify characteristic and differential fungal ASVs for the different categorical variables: microhabitat, vintage, season, *terroir*, and health type.

### Network metrics analysis

The network analysis was conducted using the R package *igraph* (version 0.10.1, Csardi and Nepusz, 2006), which provided the toolkit for our network data analysis. Raw data were transformed into adjacency matrices for subsequent statistical analysis. These matrices represented the presence or absence of ASVs for the unweighted analysis, and the abundance of different ASVs for the weighted analysis.

The function ‘graph_from_adjacency_matrix()’ from the *igraph* package was employed to convert these matrices into network objects. For the weighted network analysis, the ‘weighted’ parameter was set to true to account for the abundance values. The Louvain method was applied to identify clusters or modules within the networks. This method is suitable for both weighted and unweighted networks. The ‘cluster_louvain()’ function from *igraph* was utilized to apply the Louvain algorithm, detecting communities in each subgraph and calculating a modularity score based on these communities.

A variety of network metrics were computed for each network (weighted and unweighted). These include the average degree (using the *‘degree*()’ function), network density (using the ‘graph.density()’ function), and betweenness centrality (using the ‘betweenness()’ function). Modularity, a measure of the division strength of a network into modules, was obtained from the results of the Louvain community detection.

Custom functions and loops were designed and implemented in the code to facilitate the computation of network measures across multiple networks. These functions significantly reduced the amount of repetitive code, enhancing the efficiency and readability of the analysis. Each function was designed to accept a network object as input, perform the necessary calculation, and return the result. These functions were applied to each network in a loop, with the results compiled into a data frame for subsequent analysis.

## Results

Among the 3,010 ASVs assigned to functional groups, 733 ASVs represented 123 plant pathogenic genera, including fifteen with known associations with GTDs. The five most dominant plant pathogenic genera were: *Phaeomoniella* (80 ASVs), *Devriesia* (36 ASVs), *Fusarium* (35 ASVs), *Diplodia* (29 ASVs), and *Alternaria* (25 ASVs). Within the three different microhabitats, *Diplodia* showed the highest richness in bark and woody tissues, followed by *Phaeomoniella*, and *Devriesia*. In soil, *Fusarium* and *Alternaria* were the most diverse. The distribution and sequence read abundance of all the plant pathogenic group genera among the samples are shown in Table 2. The entire ITS rDNA region of *ca.* 800 cultures have been sequenced and more than 90% of these have also been detected by metabarcoding (Geiger et al., unpublished data).

**Table 2:** Distribution and rarefied sequence read abundance of all the plant pathogenic group genera among the samples, which were within different source types (wood, bark tissues, and soil bulk) of plant pathogenic fungal main community composition and its different *terroir*, season, year, and health.

Sample_ID	Seq. read abundance	Distribution	Microhabitat	*Terroir*	Year	Season	Health status
Sample 001	2,862	52	Bark	South	2021	Late winter	Asymptomatic
Sample 002	1,249	28	Bark	South	2021	Late winter	Symptomatic
Sample 003	2,408	34	Bark	South	2021	Late winter	Asymptomatic
Sample 004	1,293	33	Bark	South	2021	Late winter	Symptomatic
Sample 005	1,662	33	Bark	South	2021	Late winter	Asymptomatic
Sample 006	4,601	40	Bark	South	2021	Late winter	Symptomatic
Sample 007	2,156	35	Bark	South	2021	Late winter	Asymptomatic
Sample 008	2,481	23	Bark	South	2021	Late winter	Symptomatic
Sample 009	2,983	25	Bark	North	2021	Late winter	Asymptomatic
Sample 010	3,127	46	Bark	North	2021	Late winter	Symptomatic
Sample 011	3,825	49	Bark	North	2021	Late winter	Asymptomatic
Sample 012	1,533	40	Bark	North	2021	Late winter	Symptomatic
Sample 013	13,415	16	Bark	North	2021	Late winter	Asymptomatic
Sample 014	2,539	80	Bark	North	2021	Late winter	Symptomatic
Sample 015	1721	56	Bark	North	2021	Late winter	Asymptomatic
Sample 016	6,767	41	Bark	North	2021	Late winter	Symptomatic
Sample 017	2,134	48	Bark	Szt. Tamas	2021	Late winter	Asymptomatic
Sample 018	854	42	Bark	Szt. Tamas	2021	Late winter	Symptomatic
Sample 019	1,659	50	Bark	Szt. Tamas	2021	Late winter	Asymptomatic
Sample 020	587	17	Bark	Szt. Tamas	2021	Late winter	Symptomatic
Sample 021	2,539	51	Bark	Szt. Tamas	2021	Late winter	Asymptomatic
Sample 022	2,792	62	Bark	Szt. Tamas	2021	Late winter	Symptomatic
Sample 023	1,000	50	Bark	Szt. Tamas	2021	Late winter	Asymptomatic
Sample 024	1876	54	Bark	Szt. Tamas	2021	Late winter	Symptomatic
Sample 025	3,428	17	Soil	South	2021	Late winter	Asymptomatic
Sample 026	2,891	48	Soil	South	2021	Late winter	Symptomatic
Sample 027	444	2	Soil	South	2021	Late winter	Asymptomatic
Sample 028	6,014	10	Soil	South	2021	Late winter	Symptomatic
Sample 029	3,365	9	Soil	South	2021	Late winter	Asymptomatic
Sample 030	2,263	9	Soil	South	2021	Late winter	Symptomatic
Sample 031	1,383	10	Soil	South	2021	Late winter	Asymptomatic
Sample 032	1988	12	Soil	South	2021	Late winter	Symptomatic
Sample 033	1,650	39	Soil	North	2021	Late winter	Asymptomatic
Sample 034	1,434	48	Soil	North	2021	Late winter	Symptomatic
Sample 035	2,416	25	Soil	North	2021	Late winter	Asymptomatic
Sample 036	2,904	36	Soil	North	2021	Late winter	Symptomatic
Sample 037	2039	48	Soil	North	2021	Late winter	Asymptomatic
Sample 038	2,174	44	Soil	North	2021	Late winter	Symptomatic
Sample 039	1,539	54	Soil	North	2021	Late winter	Asymptomatic
Sample 040	2,388	54	Soil	North	2021	Late winter	Symptomatic
Sample 041	460	38	Soil	Szt. Tamas	2021	Late winter	Asymptomatic
Sample 042	1919	49	Soil	Szt. Tamas	2021	Late winter	Symptomatic
Sample 043	203	16	Soil	Szt. Tamas	2021	Late winter	Asymptomatic
Sample 044	989	41	Soil	Szt. Tamas	2021	Late winter	Symptomatic
Sample 045	352	37	Soil	Szt. Tamas	2021	Late winter	Asymptomatic
Sample 046	943	45	Soil	Szt. Tamas	2021	Late winter	Symptomatic
Sample 047	522	34	Soil	Szt. Tamas	2021	Late winter	Asymptomatic
Sample 048	316	28	Soil	Szt. Tamas	2021	Late winter	Symptomatic
Sample 049	7,881	26	Wood	South	2021	Late winter	Asymptomatic
Sample 050	8,898	28	Wood	South	2021	Late winter	Symptomatic
Sample 051	9,201	30	Wood	South	2021	Late winter	Asymptomatic
Sample 052	12,967	19	Wood	South	2021	Late winter	Symptomatic
Sample 053	12,734	19	Wood	South	2021	Late winter	Asymptomatic
Sample 054	3,168	28	Wood	South	2021	Late winter	Symptomatic
Sample 055	3,146	31	Wood	South	2021	Late winter	Asymptomatic
Sample 056	10,210	27	Wood	South	2021	Late winter	Symptomatic
Sample 057	2,437	46	Wood	North	2021	Late winter	Asymptomatic
Sample 058	3,231	25	Wood	North	2021	Late winter	Symptomatic
Sample 059	2013	17	Wood	North	2021	Late winter	Asymptomatic
Sample 060	8,563	37	Wood	North	2021	Late winter	Symptomatic
Sample 061	394	26	Wood	North	2021	Late winter	Asymptomatic
Sample 062	9,018	22	Wood	North	2021	Late winter	Symptomatic
Sample 063	6,134	20	Wood	North	2021	Late winter	Asymptomatic
Sample 064	8,212	10	Wood	North	2021	Late winter	Symptomatic
Sample 065	9,719	28	Wood	Szt. Tamas	2021	Late winter	Asymptomatic
Sample 066	10,277	19	Wood	Szt. Tamas	2021	Late winter	Symptomatic
Sample 067	6,939	42	Wood	Szt. Tamas	2021	Late winter	Asymptomatic
Sample 068	7,398	14	Wood	Szt. Tamas	2021	Late winter	Symptomatic
Sample 069	8,811	47	Wood	Szt. Tamas	2021	Late winter	Asymptomatic
Sample 070	4,842	18	Wood	Szt. Tamas	2021	Late winter	Symptomatic
Sample 071	736	12	Wood	Szt. Tamas	2021	Late winter	Asymptomatic
Sample 072	6,766	18	Wood	Szt. Tamas	2021	Late winter	Symptomatic
Sample 073	5,186	29	Bark	South	2021	Late summer	Asymptomatic
Sample 074	1,211	30	Bark	South	2021	Late summer	Asymptomatic
Sample 075	1,133	30	Bark	South	2021	Late summer	Symptomatic
Sample 076	290	28	Bark	South	2021	Late summer	Asymptomatic
Sample 077	2,202	45	Bark	South	2021	Late summer	Symptomatic
Sample 078	1783	31	Bark	South	2021	Late summer	Asymptomatic
Sample 079	1,333	33	Bark	South	2021	Late summer	Symptomatic
Sample 080	2,512	29	Bark	North	2021	Late summer	Asymptomatic
Sample 081	2,219	36	Bark	North	2021	Late summer	Symptomatic
Sample 082	696	27	Bark	North	2021	Late summer	Asymptomatic
Sample 083	1701	21	Bark	North	2021	Late summer	Asymptomatic
Sample 084	2,476	24	Bark	North	2021	Late summer	Symptomatic
Sample 085	3,493	42	Bark	North	2021	Late summer	Asymptomatic
Sample 086	4,073	30	Bark	North	2021	Late summer	Symptomatic
Sample 087	1,050	45	Bark	Szt. Tamas	2021	Late summer	Asymptomatic
Sample 088	1,464	29	Bark	Szt. Tamas	2021	Late summer	Symptomatic
Sample 089	481	38	Bark	Szt. Tamas	2021	Late summer	Asymptomatic
Sample 090	1983	42	Bark	Szt. Tamas	2021	Late summer	Symptomatic
Sample 091	1,601	40	Bark	Szt. Tamas	2021	Late summer	Asymptomatic
Sample 092	913	11	Bark	Szt. Tamas	2021	Late summer	Symptomatic
Sample 093	1,556	44	Bark	Szt. Tamas	2021	Late summer	Asymptomatic
Sample 094	6,143	36	Bark	Szt. Tamas	2021	Late summer	Symptomatic
Sample 095	1,514	7	Soil	South	2021	Late summer	Asymptomatic
Sample 096	5,013	26	Soil	South	2021	Late summer	Symptomatic
Sample 097	6,389	4	Soil	South	2021	Late summer	Asymptomatic
Sample 098	4,323	7	Soil	South	2021	Late summer	Symptomatic
Sample 099	1,250	9	Soil	South	2021	Late summer	Asymptomatic
Sample 100	3,656	16	Soil	South	2021	Late summer	Symptomatic
Sample 101	2,447	32	Soil	South	2021	Late summer	Asymptomatic
Sample 102	1,511	11	Soil	South	2021	Late summer	Symptomatic
Sample 103	1,519	33	Soil	North	2021	Late summer	Asymptomatic
Sample 104	2082	41	Soil	North	2021	Late summer	Symptomatic
Sample 105	1,582	35	Soil	North	2021	Late summer	Asymptomatic
Sample 106	1776	41	Soil	North	2021	Late summer	Symptomatic
Sample 107	1,345	43	Soil	North	2021	Late summer	Asymptomatic
Sample 108	1,328	26	Soil	North	2021	Late summer	Symptomatic
Sample 109	1,148	33	Soil	North	2021	Late summer	Asymptomatic
Sample 110	1,373	46	Soil	North	2021	Late summer	Symptomatic
Sample 111	682	32	Soil	Szt. Tamas	2021	Late summer	Asymptomatic
Sample 112	1,186	50	Soil	Szt. Tamas	2021	Late summer	Symptomatic
Sample 113	572	43	Soil	Szt. Tamas	2021	Late summer	Asymptomatic
Sample 114	1,610	45	Soil	Szt. Tamas	2021	Late summer	Symptomatic
Sample 115	743	38	Soil	Szt. Tamas	2021	Late summer	Asymptomatic
Sample 116	808	43	Soil	Szt. Tamas	2021	Late summer	Symptomatic
Sample 117	1,381	54	Soil	Szt. Tamas	2021	Late summer	Asymptomatic
Sample 118	1,417	31	Soil	Szt. Tamas	2021	Late summer	Symptomatic
Sample 119	10,269	19	Wood	South	2021	Late summer	Asymptomatic
Sample 120	14,490	17	Wood	South	2021	Late summer	Symptomatic
Sample 121	11,596	27	Wood	South	2021	Late summer	Symptomatic
Sample 122	6,330	23	Wood	South	2021	Late summer	Asymptomatic
Sample 123	9,921	23	Wood	South	2021	Late summer	Symptomatic
Sample 124	10,624	19	Wood	South	2021	Late summer	Asymptomatic
Sample 125	5,033	21	Wood	South	2021	Late summer	Symptomatic
Sample 126	2,506	21	Wood	North	2021	Late summer	Asymptomatic
Sample 127	9,935	25	Wood	North	2021	Late summer	Symptomatic
Sample 128	1,151	23	Wood	North	2021	Late summer	Asymptomatic
Sample 129	6,448	37	Wood	North	2021	Late summer	Symptomatic
Sample 130	5,582	31	Wood	North	2021	Late summer	Asymptomatic
Sample 131	1,617	21	Wood	North	2021	Late summer	Symptomatic
Sample 132	12,034	32	Wood	North	2021	Late summer	Symptomatic
Sample 133	6,875	27	Wood	Szt. Tamas	2021	Late summer	Asymptomatic
Sample 134	8,151	21	Wood	Szt. Tamas	2021	Late summer	Symptomatic
Sample 135	14,452	20	Wood	Szt. Tamas	2021	Late summer	Asymptomatic
Sample 136	5,885	12	Wood	Szt. Tamas	2021	Late summer	Symptomatic
Sample 137	12,755	46	Wood	Szt. Tamas	2021	Late summer	Asymptomatic
Sample 138	14,410	50	Wood	Szt. Tamas	2021	Late summer	Symptomatic
Sample 139	13,126	18	Wood	Szt. Tamas	2021	Late summer	Asymptomatic
Sample 140	9,521	36	Wood	Szt. Tamas	2021	Late summer	Symptomatic
Sample 141	7,615	52	Bark	South	2020	Late summer	Asymptomatic
Sample 142	3,393	19	Bark	South	2020	Late winter	Asymptomatic
Sample 143	7,063	45	Bark	South	2020	Late summer	Symptomatic
Sample 144	3,428	22	Bark	South	2020	Late winter	Symptomatic
Sample 145	1851	39	Bark	South	2020	Late summer	Asymptomatic
Sample 146	2,584	32	Bark	South	2020	Late winter	Asymptomatic
Sample 147	3,611	47	Bark	South	2020	Late summer	Symptomatic
Sample 148	3,708	23	Bark	South	2020	Late winter	Symptomatic
Sample 149	2,262	22	Bark	South	2020	Late winter	Asymptomatic
Sample 150	857	47	Bark	South	2020	Late summer	Symptomatic
Sample 151	3,461	28	Bark	South	2020	Late winter	Symptomatic
Sample 152	516	35	Bark	South	2020	Late summer	Asymptomatic
Sample 153	833	27	Bark	South	2020	Late winter	Asymptomatic
Sample 154	4,551	38	Bark	South	2020	Late summer	Symptomatic
Sample 155	2,162	34	Bark	South	2020	Late winter	Symptomatic
Sample 156	1,228	35	Bark	North	2020	Late summer	Asymptomatic
Sample 157	3,183	26	Bark	North	2020	Late winter	Asymptomatic
Sample 158	1787	32	Bark	North	2020	Late summer	Symptomatic
Sample 159	4,047	29	Bark	North	2020	Late winter	Symptomatic
Sample 160	325	27	Bark	North	2020	Late summer	Asymptomatic
Sample 161	1,082	17	Bark	North	2020	Late winter	Asymptomatic
Sample 162	1,477	28	Bark	North	2020	Late summer	Symptomatic
Sample 163	2,256	17	Bark	North	2020	Late winter	Symptomatic
Sample 164	1,224	63	Bark	North	2020	Late summer	Asymptomatic
Sample 165	3,064	32	Bark	North	2020	Late winter	Asymptomatic
Sample 166	4,355	39	Bark	North	2020	Late summer	Symptomatic
Sample 167	3,994	23	Bark	North	2020	Late winter	Symptomatic
Sample 168	2,155	42	Bark	North	2020	Late summer	Asymptomatic
Sample 169	1,495	32	Bark	North	2020	Late winter	Asymptomatic
Sample 170	1741	41	Bark	North	2020	Late summer	Symptomatic
Sample 171	3,787	35	Bark	North	2020	Late winter	Symptomatic
Sample 172	880	57	Bark	Szt. Tamas	2020	Late summer	Asymptomatic
Sample 173	836	35	Bark	Szt. Tamas	2020	Late winter	Asymptomatic
Sample 174	980	31	Bark	Szt. Tamas	2020	Late summer	Symptomatic
Sample 175	3,607	23	Bark	Szt. Tamas	2020	Late winter	Symptomatic
Sample 176	4,231	57	Bark	Szt. Tamas	2020	Late summer	Asymptomatic
Sample 177	855	34	Bark	Szt. Tamas	2020	Late winter	Asymptomatic
Sample 178	437	47	Bark	Szt. Tamas	2020	Late summer	Symptomatic
Sample 179	491	26	Bark	Szt. Tamas	2020	Late winter	Symptomatic
Sample 180	1,054	27	Bark	Szt. Tamas	2020	Late summer	Asymptomatic
Sample 181	314	12	Bark	Szt. Tamas	2020	Late winter	Asymptomatic
Sample 182	693	49	Bark	Szt. Tamas	2020	Late summer	Symptomatic
Sample 183	3,475	30	Bark	Szt. Tamas	2020	Late winter	Symptomatic
Sample 184	1,140	42	Bark	Szt. Tamas	2020	Late summer	Asymptomatic
Sample 185	438	24	Bark	Szt. Tamas	2020	Late winter	Asymptomatic
Sample 186	5,682	51	Bark	Szt. Tamas	2020	Late summer	Symptomatic
Sample 187	478	27	Bark	Szt. Tamas	2020	Late winter	Symptomatic
Sample 188	2,667	86	Soil	South	2020	Late summer	Asymptomatic
Sample 189	2,191	49	Soil	South	2020	Late winter	Asymptomatic
Sample 190	3,778	102	Soil	South	2020	Late summer	Symptomatic
Sample 191	3,104	51	Soil	South	2020	Late winter	Symptomatic
Sample 192	2,230	69	Soil	South	2020	Late summer	Asymptomatic
Sample 193	1891	43	Soil	South	2020	Late winter	Asymptomatic
Sample 194	2,830	74	Soil	South	2020	Late summer	Symptomatic
Sample 195	1767	42	Soil	South	2020	Late winter	Symptomatic
Sample 196	1,183	71	Soil	South	2020	Late summer	Asymptomatic
Sample 197	1,541	35	Soil	South	2020	Late winter	Asymptomatic
Sample 198	2,638	83	Soil	South	2020	Late summer	Symptomatic
Sample 199	7,508	41	Soil	South	2020	Late winter	Symptomatic
Sample 200	2,553	100	Soil	South	2020	Late summer	Asymptomatic
Sample 201	2,986	98	Soil	South	2020	Late summer	Symptomatic
Sample 202	186	12	Soil	South	2020	**Late winter**	**Asymptomatic**
Sample 203	4,073	47	Soil	South	2020	Late winter	Symptomatic
Sample 204	1,355	60	Soil	North	2020	Late summer	Asymptomatic
Sample 205	1,577	36	Soil	North	2020	Late winter	Asymptomatic
Sample 206	1,687	84	Soil	North	2020	Late summer	Symptomatic
Sample 207	926	39	Soil	North	2020	Late winter	Symptomatic
Sample 208	1,508	76	Soil	North	2020	Late summer	Asymptomatic
Sample 209	1895	51	Soil	North	2020	Late winter	Asymptomatic
Sample 210	1,611	82	Soil	North	2020	Late summer	Symptomatic
Sample 211	3,793	37	Soil	North	2020	Late winter	Symptomatic
Sample 212	1,396	64	Soil	North	2020	Late summer	Asymptomatic
Sample 213	1,105	41	Soil	North	2020	Late winter	Asymptomatic
Sample 214	1823	76	Soil	North	2020	Late summer	Symptomatic
Sample 215	1,389	46	Soil	North	2020	Late winter	Symptomatic
Sample 216	1,507	78	Soil	North	2020	Late summer	Asymptomatic
Sample 217	852	48	Soil	North	2020	Late winter	Asymptomatic
Sample 218	1,053	60	Soil	North	2020	Late summer	Symptomatic
Sample 219	2,594	42	Soil	North	2020	Late winter	Symptomatic
Sample 220	2,328	77	Soil	Szt. Tamas	2020	Late summer	Asymptomatic
Sample 221	1,523	36	Soil	Szt. Tamas	2020	Late winter	Asymptomatic
Sample 222	4,047	96	Soil	Szt. Tamas	2020	Late summer	Symptomatic
Sample 223	2,981	40	Soil	Szt. Tamas	2020	Late winter	Symptomatic
Sample 224	1,095	89	Soil	Szt. Tamas	2020	Late summer	Asymptomatic
Sample 225	661	43	Soil	Szt. Tamas	2020	Late winter	Asymptomatic
Sample 226	1767	118	Soil	Szt. Tamas	2020	Late summer	Symptomatic
Sample 227	3,711	51	Soil	Szt. Tamas	2020	Late winter	Symptomatic
Sample 228	565	77	Soil	Szt. Tamas	2020	Late summer	Asymptomatic
Sample 229	991	32	Soil	Szt. Tamas	2020	Late winter	Asymptomatic
Sample 230	1,548	69	Soil	Szt. Tamas	2020	Late summer	Symptomatic
Sample 231	737	32	Soil	Szt. Tamas	2020	Late winter	Symptomatic
Sample 232	522	57	Soil	Szt. Tamas	2020	Late summer	Asymptomatic
Sample 233	351	24	Soil	Szt. Tamas	2020	Late winter	Asymptomatic
Sample 234	1721	58	Soil	Szt. Tamas	2020	Late summer	Symptomatic
Sample 235	554	30	Soil	Szt. Tamas	2020	Late winter	Symptomatic
Sample 236	10,210	44	Wood	South	2020	Late summer	Asymptomatic
Sample 237	5,121	44	Wood	South	2020	Late winter	Asymptomatic
Sample 238	11,375	53	Wood	South	2020	Late summer	Symptomatic
Sample 239	6,570	15	Wood	South	2020	Late winter	**Symptomatic**
Sample 240	4,194	65	Wood	South	2020	Late summer	Asymptomatic
Sample 241	13,001	50	Wood	South	2020	Late winter	Asymptomatic
Sample 242	6,718	57	Wood	South	2020	Late summer	Symptomatic
Sample 243	8,058	36	Wood	South	2020	Late winter	Symptomatic
Sample 244	11,230	55	Wood	South	2020	Late winter	Asymptomatic
Sample 245	12,754	75	Wood	South	2020	Late summer	Symptomatic
Sample 246	15,113	35	Wood	South	2020	Late summer	Asymptomatic
Sample 247	12,384	37	Wood	South	2020	Late winter	Symptomatic
Sample 248	2,388	29	Wood	North	2020	Late summer	Asymptomatic
Sample 249	7,604	33	Wood	North	2020	Late winter	Asymptomatic
Sample 250	8,518	36	Wood	North	2020	Late summer	Symptomatic
Sample 251	10,026	30	Wood	North	2020	Late winter	Symptomatic
Sample 252	8,871	55	Wood	North	2020	Late summer	Asymptomatic
Sample 253	1,568	32	Wood	North	2020	Late winter	Asymptomatic
Sample 254	7,772	52	Wood	North	2020	Late winter	Symptomatic
Sample 255	4,222	52	Wood	North	2020	Late summer	Asymptomatic
Sample 256	4,826	23	Wood	North	2020	Late winter	Asymptomatic
Sample 257	12,372	20	Wood	North	2020	Late summer	Symptomatic
Sample 258	5,007	20	Wood	North	2020	Late winter	Symptomatic
Sample 259	7,930	43	Wood	North	2020	Late summer	Asymptomatic
Sample 260	1984	38	Wood	North	2020	Late winter	Asymptomatic
Sample 261	2049	51	Wood	North	2020	Late summer	Symptomatic
Sample 262	9,176	29	Wood	North	2020	Late winter	Symptomatic
Sample 263	3,286	53	Wood	Szt. Tamas	2020	Late summer	Asymptomatic
Sample 264	6,594	34	Wood	Szt. Tamas	2020	Late winter	Asymptomatic
Sample 265	10,868	32	Wood	Szt. Tamas	2020	Late summer	Symptomatic
Sample 266	4,883	24	Wood	Szt. Tamas	2020	Late winter	Symptomatic
Sample 267	11,845	55	Wood	Szt. Tamas	2020	Late summer	Asymptomatic
Sample 268	10,514	38	Wood	Szt. Tamas	2020	Late winter	Asymptomatic
Sample 269	4,455	48	Wood	Szt. Tamas	2020	Late summer	Symptomatic
Sample 270	2,960	20	Wood	Szt. Tamas	2020	Late winter	Symptomatic
Sample 271	9,240	50	Wood	Szt. Tamas	2020	Late summer	Asymptomatic
Sample 272	10,753	30	Wood	Szt. Tamas	2020	Late winter	Asymptomatic
Sample 273	7,842	40	Wood	Szt. Tamas	2020	Late summer	Symptomatic
Sample 274	9,837	30	Wood	Szt. Tamas	2020	Late winter	Symptomatic
Sample 275	8,866	42	Wood	Szt. Tamas	2020	Late summer	Asymptomatic
Sample 276	11,692	29	Wood	Szt. Tamas	2020	Late winter	Asymptomatic
Sample 277	9,449	35	Wood	Szt. Tamas	2020	Late summer	Symptomatic
Sample 278	8,137	44	Wood	Szt. Tamas	2020	Late winter	Symptomatic

GTDs represent Grapevine Trunk Diseases representatives’ genera.

The variance analyses for richness and abundance values of different plant pathogens across different microhabitats (sources), *terroirs*, vintage, season, and health types revealed notable distinctions. For plant pathogenic fungi, richness exhibited significant differences among microhabitats/sources (*p* = 7.64 × 10^−8^), vintage (*p* = 1.74 × 10^−14^), and season (*p* = 2.79 × 10^−11^). However, the *terroir* (*p* = 0.168) and health types (*p* = 0.242) did not significantly impact richness. Regarding rarefied fungal read abundance values of plant pathogenic fungi, significant differences were observed among different microhabitats (*p* = 2×10^−16^), *terroirs* (*p* = 0.000168), and health types (*p* = 0.033922). Meanwhile, the influence of vintage (*p* = 0.785) and season (*p* = 0.363) on abundance was not significant. Additionally, total richness values were significantly different for vintage (*p* = 2.60 × 10^−14^) and season (*p* = 2.32 × 10^−11^), whereas abundance values showed significant differences with *terroirs* (*p* = 7.86 × 10^−4^) and health types (*p* = 0.033922).

**Figure 1:**
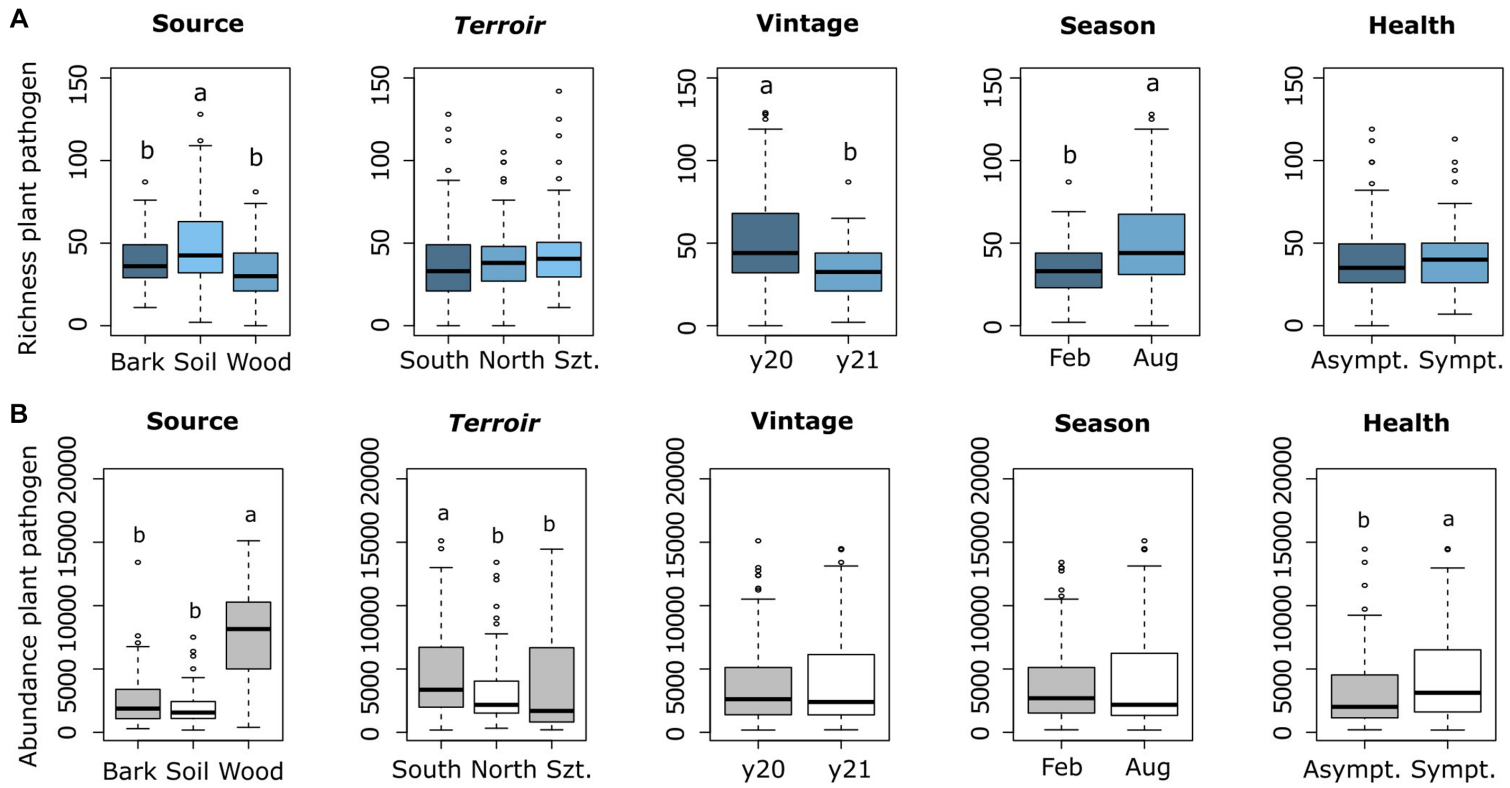
Comparison of plant pathogenic richness **(A)** and rarefied fungal read abundance **(B)** across the four-parameters and source types, which represented as Szt (Szt. Tamás *terroir*), y20 (year 2020), y21 (year 2021), Feb (February), and Aug (August). Means were compared using ANOVA and Tukey’s HSD tests, with letters denoting significant differences.

The fungal richness was greatest in soil, followed by bark and wood (Figure 1A). In terms of abundance values, wood showed by far the highest amount, followed by bark and soil (Figure 1B). Plant pathogens abundance considering health types was highest in symptomatic plants (Sympt.) compared to non-symptomatic (Asympt.) (Figure 1B). Another observation was the highest richness in August (late summer) compared to February (late winter) in the seasonality parameter, while observed richness in 2020 was higher than in 2021 (Figure 1A).

**Figure 2:**
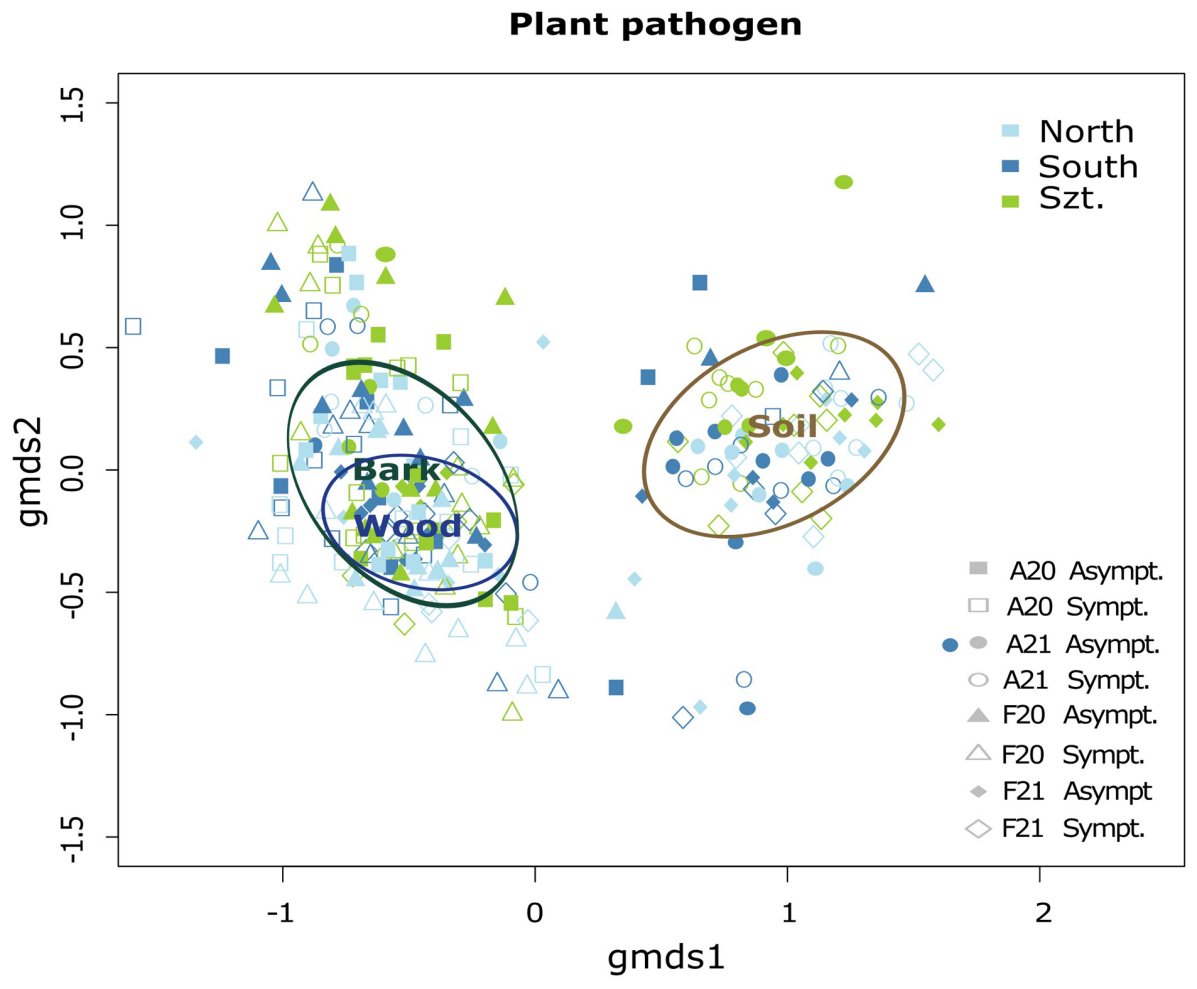
Two-dimensional non-metric multidimensional scaling (NMDS) ordination illustrating the combined community structure of plant pathogenic fungi with ellipses representing the ordination scalability of the microhabitats: wood, bark, and soil. The overall stress value for this combined analysis is 0.07869204.

**Figure 3:**
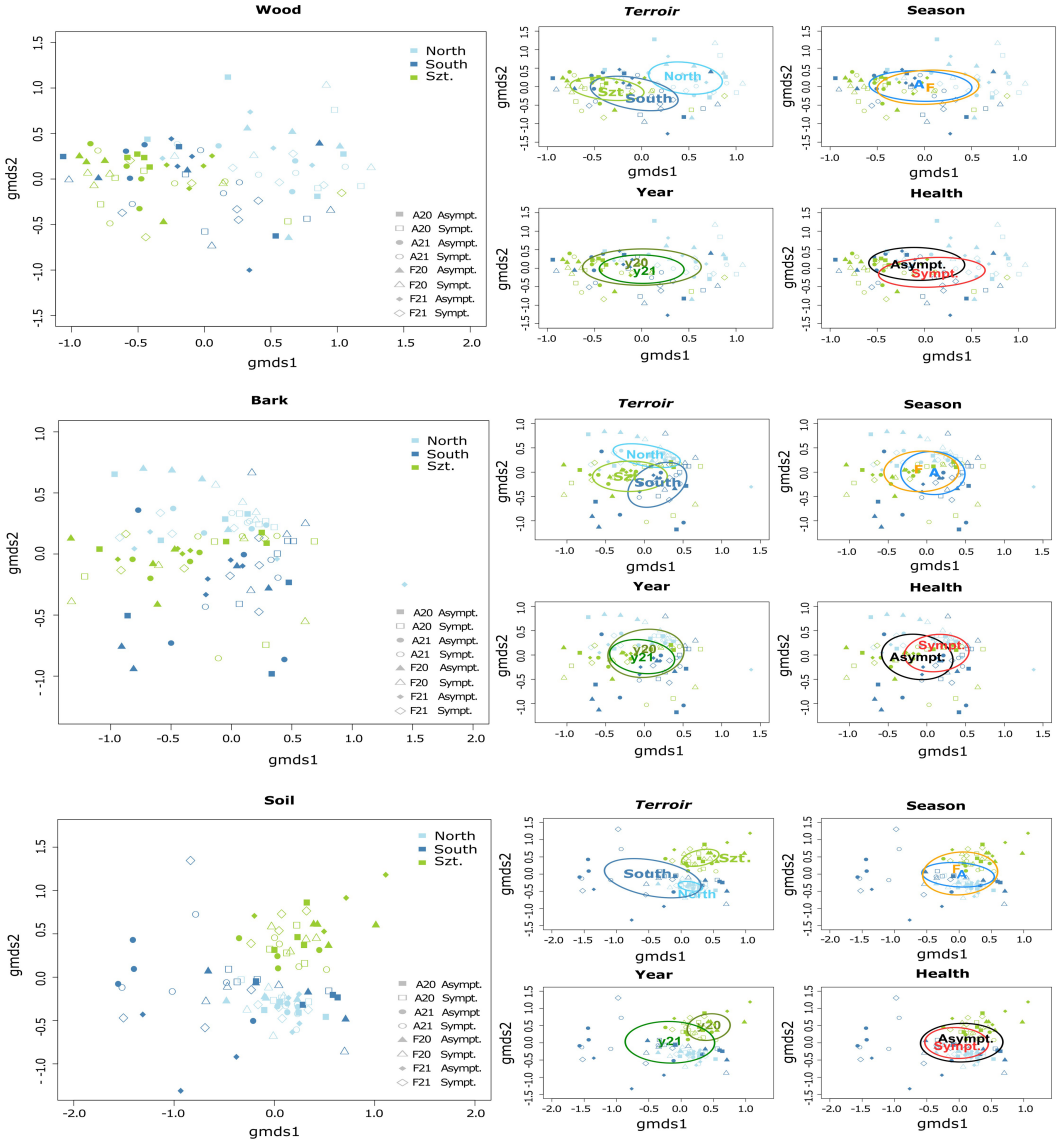
Separate two-dimensional non-metric multidimensional scaling (NMDS) ordinations representing the community structure of plant pathogenic fungi in each microhabitat: wood, bark, and soil. Each graph demonstrates variations according to different parameters, namely *terroir* (North, South, and Szt. Tamás), health type (Asymptomatic and Symptomatic), vintage (20 for year 2020, and 21 for year 2021), and season (A = August/Late summer, and F = February/Late winter). Individual stress values for each microhabitat analysis are as follows: wood = 0.08765602, bark = 0.09239610, and soil = 0.07753184.

Fungal community composition was visualized in a two-dimensional NMDS ordination for all the plant pathogenic community (stress value = 0.07869204), as well as each source: wood (stress value = 0.08765602), bark (stress value = 0.09239610) and soil (stress value = 0.07753184) (Figure 2). PERMANOVA, using *adonis,* indicated that the composition was strongly correlated with *terroirs*, vintage, and season, in this order of strength, for all microhabitats, while significant compositional difference of fungi between asymptomatic and symptomatic grapevines was only observed in bark and inner woody tissue (Figure 3; Table 3). Although fungi from all three microhabitats showed compositional differences among *terroirs*, soil communities showed the greatest effect of *terroir* on compositional turnover, explaining 24.67% of variance (*p* = 0.0001), followed by bark (16.56%, *p* = 0.0001) and woody tissue (14.46%, *p* = 0.0001). The effect of vintage showed the same above pattern as *terroir*, although it was much weaker, while the influence of season was greatest on bark communities (2.98%, *p* = 0.0002), followed by soil (1.93%, *p* = 0.0036), and wood (1.84%, *p* = 0.0337). As for vintage, it has the same tendency as *terroir*. Health type only explained *ca.* 3% of the compositional variance in woody tissue and bark (Table 3).

**Table 3:** Proportion of variation (%) in different source types (wood, bark tissues and soil bulk) of fungal community composition explained by different *terroir*, season, year, and health calculated with permutational multivariate analysis of variance, based on the fungal community matrix.

	*Terroir*		Season		Vintage		Health	
	%	*p* value	%	*p* value	%	*p* value	%	*p* value
All	1.825	**0.0002**	0.872	**0.0066**	9.034	**0.0001**	0.186	0.8967
Wood	14.46	**0.0001**	1.842	**0.0337**	3.675	**0.001**	3.329	**0.0007**
Bark	16.56	**0.0001**	2.984	**0.0002**	4.535	**0.0003**	3.535	**0.0001**
Soil	24.673	**0.0001**	1.928	**0.0036**	6.388	**0.0001**	1.105	0.0973

Followed by significant *p* values from variance analysis among the taxonomic groups regarding richness. Significant results are in bold.

The assessment of climatic variables, temperature and precipitation, displayed significant influence on fungal community composition (Table 1). Both seasonal and vintage parameters contributed to the variation in fungal communities. The late winter season exhibited a statistically significant impact on fungal community composition, explaining 1.188% of the variance (*p* = 0.0012) in temperature. Similar to late winter, the late summer season significantly contributed to compositional changes, accounting for 1.179% of the variance (*p* = 0.0011). This underscores the sensitivity of fungal communities to temperature fluctuations during the colder and warmer months. In contrast to temperature, precipitation during late winter did not show a significant impact on fungal communities (0.239%, *p* = 0.7607). Late summer precipitation, on the other hand, played a more substantial role, explaining 1.074% of the variance (*p* = 0.0031).

**Figure 4:**
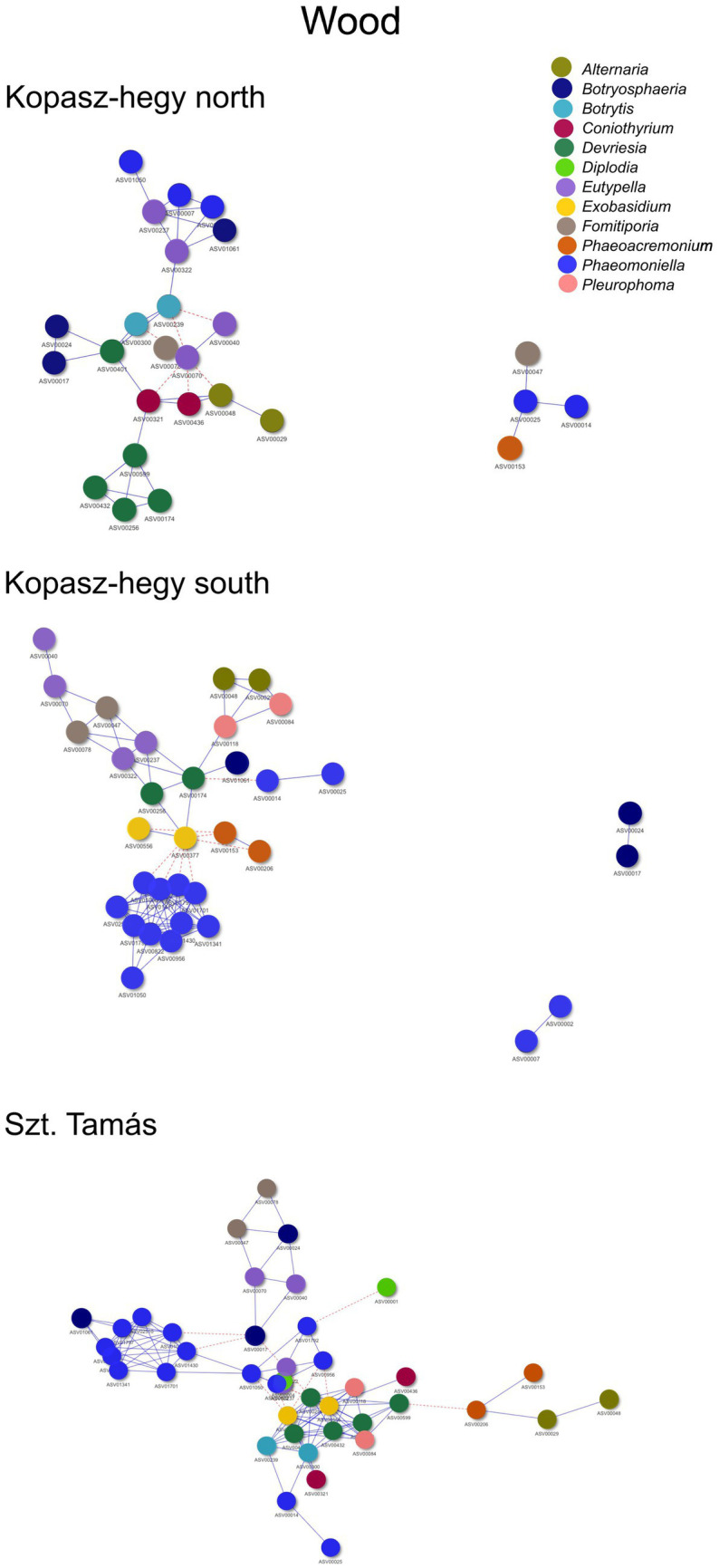
Cooccurrence comparison visual network of the microhabitat wood visualized by the method ‘*visNetwork’*. Nodes represented by each specific ASVs with positive cooccurrence being represented by the blue lines and red dotted lines the negatives. A follow up table with specificities (descriptive statistics) can be seen in Supplementary Table S1. Legend specifies the main important genus represented by ASVs shown in the network.

**Figure 5:**
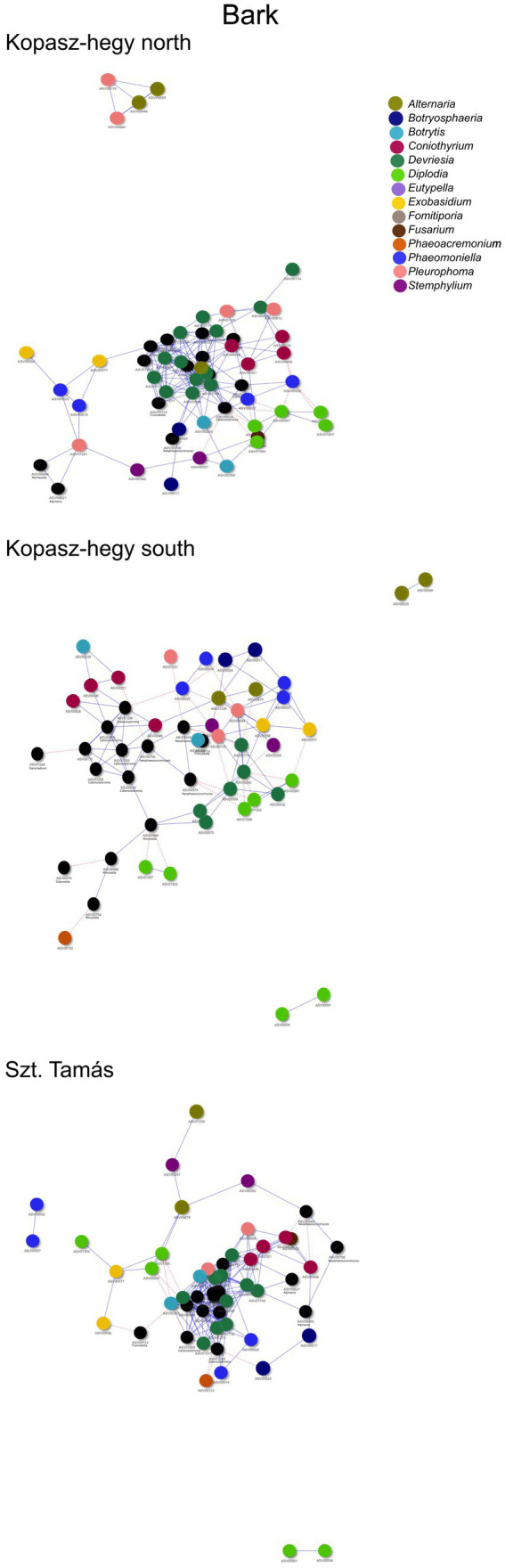
Visual network comparing the cooccurrence of microhabitat in Bark ASVs using the *‘visNetwork’* method. Nodes represent individual ASVs, with blue lines indicating positive cooccurrence and red dotted lines indicating negative cooccurrence. A table with descriptive statistics can be found in Supplementary Table S1. The legend highlights the main genus represented by ASVs in the network.

**Figure 6:**
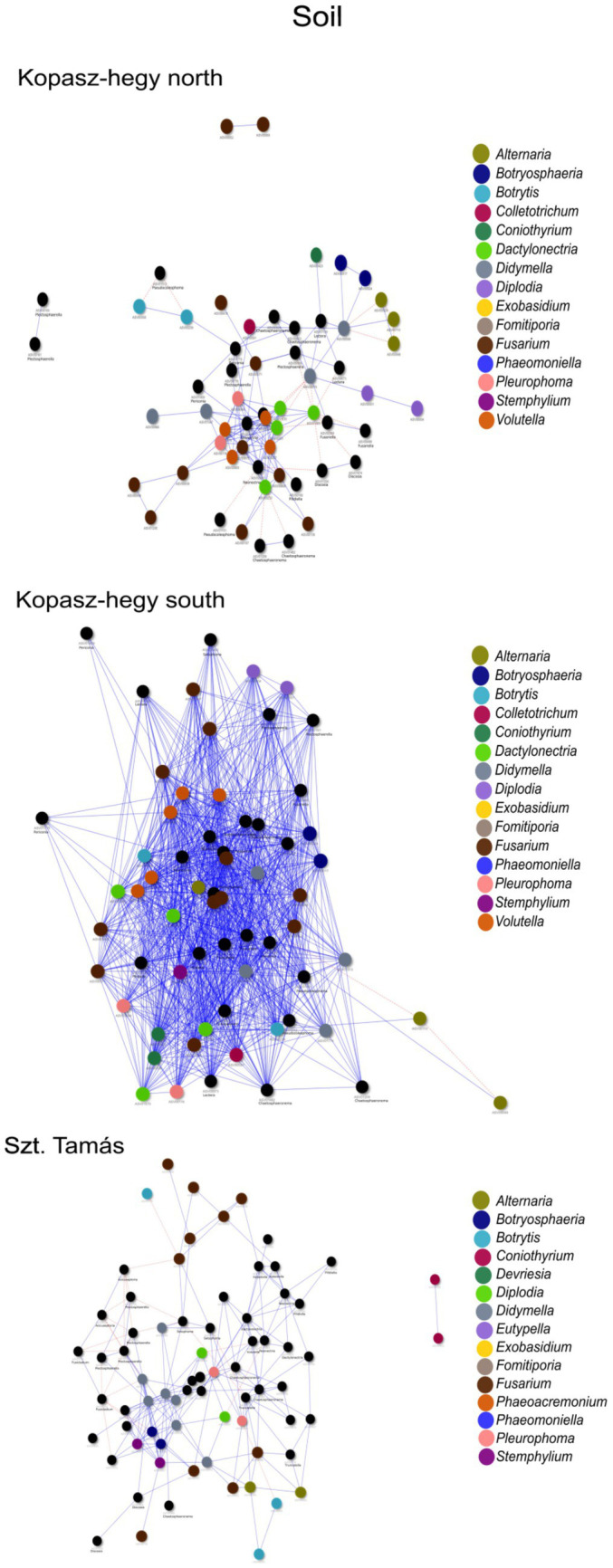
Network visualization comparing the co-occurrence of microhabitat soil ASVs using the *‘visNetwork’* method. Each node represents a specific ASV, and the blue lines indicate positive co-occurrence while the red dotted lines indicate negative co-occurrence. Supplementary Table S1 provides additional descriptive statistics. The legend indicates the key genus represented by ASVs in the network with rendered imaging for size fitting of the visualization.

The year 2020 contributed significantly to the variance in fungal communities, with temperature explaining 1.09% (*p* = 0.0021) and precipitation explaining 2.215% (*p* = 0.0001). In 2021, the impact of temperature reduced to 0.646% (*p* = 0.0451), and precipitation explained 0.781% of the variance (*p* = 0.035). Although less pronounced, these results indicate continued but potentially attenuated seasonal effects on fungal communities in the subsequent year. Cooccurrence analyses and network graphs revealed differences in network complexity of plant pathogenic fungi among microhabitats in each *terroir*. The size of networks of significant relationships increased from woody tissue (265 ASVs) to bark (566 ASVs) and to soil (1,669 ASVs) (Figures 4–6; Supplementary Table S1). Positive relationships were more prevalent than negative ones in all microhabitats, as evidenced by the co-occurrence analysis (Figures 4–6). In soil (Figure 6), there were 1,407 positive and 262 negative relationships between pairs of ASVs, in bark (Figure 5) there were 414 positive and 152 negative relationships, and in wood (Figure 4), there were 195 positive and 70 negative relationships, respectively.

The network metrics analysis elucidated the structure of microbial communities across different conditions, focusing on the specific metrics of betweenness and degree for the weighted analysis, and average degree, network density, and modularity for the unweighted analysis (Supplementary Tables S2, S3). Considering the abundance of different ASVs, the weighted network analysis showed from the weighted network metrics that for the bark microhabitat during late winter in vintage year ‘21’, the southern *terroir* showed the highest degree (3.662650602) for asymptomatic health status. Interestingly, the bark microhabitat in the northern *terroir* demonstrated the highest betweenness (147.316092) for symptomatic health status (Supplementary Table S2).

However, the soil microhabitat during late summer of vintage year ‘20’, the southern *terroir* showed the highest degree (4.418918919) for symptomatic health status, while the soil northern *terroir* during late summer of the same year displayed the highest betweenness (150.5374717) for symptomatic health status (Supplementary Table S2). As for the wood microhabitat during late winter in vintage year ‘21’, the southern *terroir* exhibited the highest degree (3.672727273) for symptomatic health status, while the northern *terroir*, late winter of the same year, demonstrated the highest betweenness (120.4482759) (Supplementary Table S2) for symptomatic health status.

The unweighted network analysis, considering the presence or absence of different ASVs, revealed for the bark microhabitat during late winter of vintage year ‘21’, the southern *terroir* demonstrated the highest average degree (3.69047619) with a network density of 0.044463569 and a modularity of 0.266118626 for asymptomatic health status (Supplementary Table S3). In the soil microhabitat during late winter in vintage year ‘21’, the southern *terroir* had the highest network density (0.071969697) with an average degree of 2.303030303 and a modularity of 0.457063712 for asymptomatic health status. For the wood microhabitat during late winter in vintage year ‘21’, the southern *terroir* showed the highest average degree (3.593220339) with a network density of 0.061952075 and a modularity of 0.268823425 for symptomatic health status (Supplementary Table S3).

Collectively, the network structures of the fungal pathobiome differ significantly among the grapevine microhabitats, with intricate and robust network structures evident in certain conditions.

The number of indicator species was the highest in the soil, representing the genera *Fusarium*, *Lactera*, *Alternaria*, *Coniothyrium*, *Dactylonectria*, *Plectosphaerella*, *Botryosphaeria* etc. (Supplementary Table S4). The most significant indicators of *terroir* in soil were *Lectera* (North), *Alternaria* (South), and *Coniothyrium* (Szt. Tamás). For season, the most significant indicator was *Fusarium* (late summer), while none of the ASVs was an indicator for late winter. Lastly, the strongest indicators for vintage in soil were *Dactylonectria* (2020) and *Plectosphaerella* (2021). In bark samples, *Diplodia*, *Microstroma*, *Devriesia*, *Botrytis* was the most significant identified as indicators among the parameters, with an especially high presence of the genus *Diplodia*. In wood, significant indicators mostly belonged to grapevine trunk disease, such as *Phaeomoninella*, *Diplodia*, and *Eutypella*. From those, the genus *Diplodia* and *Eutypella* were strongly significant to the symptomatic of the health type parameter, and *Devriesia* and *Phaeomoniella* to the asymptomatic (Supplementary Table S2). Interestingly, in wood *terroir* the genus *Pleurophoma* and *Diplodia* were most significant for North and South, but in Szt. Tamás there were only indicators of the genus *Phaeomoniella*.

## Discussion

This study delves into the intricate structure and dynamics of the grapevine pathobiome, primarily focusing on how microhabitat, *terroir*, vintage, season, and health status shape these complex fungal communities. In alignment with our first hypothesis, our findings illustrate a significant variation in richness and composition of plant pathogenic fungi among different microhabitats. Specifically, a decrease in the richness of plant pathogenic fungi was observed from soil to bark and wood samples, while the richness of pathogens associated with GTDs followed an increasing trend. This reinforces the concept of niche specialization within these fungal communities, demonstrating that certain microhabitats favor the proliferation of specific fungal groups. In addition, our findings suggest that selection pressure of abiotic environmental factors is buffered to a certain extent inside the grapevine trunk, as indicated by the somewhat weaker, although still significant, effects of *terroir*, season, and vintage on fungal community composition in wood, compared to bark and soil communities.

The soil, known to be greatly influenced by both mesoclimatic and edaphic factors worldwide (Tedersoo et al., 2014; Větrovský et al., 2019) as well as in the wider region of study in northeastern Hungary (Geml, 2019; Geml et al., 2022), exhibited the highest fungal richness, particularly during the late summer.

Adding depth to our understanding, the indicator species analysis shed light on specific fungal taxa serving as microbial fingerprints across various microhabitats. For instance, in the soil, where NMDS highlighted vintage influences, we found *Fusarium* indicative of late summer and *terroir*-specific markers such as *Lactera* (North) and *Alternaria* (South). Bark samples pointed toward *Diplodia*’s prominence, and in the wood, disease-associated genera like *Phaeomoninella* and *Diplodia* stood out, especially in relation to plant health status. These indicator species, in harmony with NMDS findings, emphasize the intricate connections between fungal communities, their microhabitats, and external factors.

The isolation of fungi from inner wood samples has provided additional insights into the pathobiome related to GTDs. More than 90% of the fungal genera detected through metabarcoding were also isolated from inner wood samples, confirming their presence within the grapevine (Geiger et al., unpublished data). This suggests that these genera are well-established members of the grapevine pathobiome and play a significant role in grapevine health and disease development.

Building upon this, it’s important to delve deeper into the nuances of vintage variations. In terms of vintage, distinct patterns were observed across different microhabitats. The NMDS ordination (Figure 3) illustrates a clearer separation of fungal communities between the years 2020 and 2021 in the soil samples, indicating a notable impact of vintage in this microhabitat. The year 2020 exhibited higher fungal richness compared to 2021. This difference could be attributed to the distinct climatic conditions each year presented, with 2020 having an early and extended rainy period, whereas 2021 experienced a severe heatwave and drought, as reported by the Copernicus Climate Change Service (C3S) and the European State of Climate (ESOTC).

These adverse weather conditions created significant mesoclimatic differences and could have led to a temporary decrease in overall plant pathogenic fungal diversity. This likely is the result of a selection pressure favoring taxa that can withstand low moisture content and adapt to shifts in the diffusion rates of nutrients and microbial signals. Many of these microbial signals, such as mycotoxins, volatile organic compounds (VOCs), and extracellular enzymes, are influenced by environmental moisture content (Henry et al., 2007; Smith and De Smet, 2012; Song et al., 2012; Barnard et al., 2013; Bokulich et al., 2014; Naylor et al., 2017; Santos-Medellín et al., 2017; Naylor and Coleman-Derr, 2018). Under drier conditions, the diffusion of these signals in the soil might be hindered, affecting both fungal-fungal and plant-fungal interactions. This can modify competition dynamics, nutrient acquisition strategies, and even the establishment of symbiotic relationships.

Nevertheless, the highest rarefied fungal read abundance of plant pathogens was observed in wood and in symptomatic plants, underscoring that both microhabitat and health status exert a significant influence on the diversity and abundance of fungal communities. Our second hypothesis suggested that the diversity, abundance, and distribution of the pathobiome are influenced by different parameters, such as *terroir*, seasonality, and vintage aspects. In support of this hypothesis, we found that compositional shifts in these fungal communities were chiefly influenced by site-specific environmental factors, often referred to as “microbial *terroir*.” These observations align with previous research indicating that both mesoclimatic and edaphic factors substantially shape fungal communities worldwide (Tedersoo et al., 2014; Větrovský et al., 2019). It becomes increasingly apparent that unraveling the intricate relationships between different microhabitats and their unique ‘microbial *terroir’* characteristics is essential for comprehending the nuanced composition of the pathobiome.

Moreover, our investigation revealed a notable influence of vintage and seasonality on the pathobiome. In terms of richness and abundance, the soil and late summer season presented the highest fungal richness, while wood and symptomatic plants exhibited the highest abundance. These patterns suggest that both environmental conditions and plant health status can significantly influence the diversity and abundance of fungal communities. The NMDS ordination further substantiated these patterns, revealing distinct clustering of fungal communities based on *terroir*, vintage, and season, thereby affirming a strong influence of these factors on community composition.

The integration of climatic variables, temperature, and precipitation into our analysis provides a nuanced understanding of their impact on fungal community composition across seasons and vintages. Late winter and late summer exerted distinct influences, with temperature changes during these seasons significantly contributing to the variance. Interestingly, late summer precipitation played a more substantial role than its winter counterpart, underlining the sensitivity of fungal communities to warmer and drier conditions. Vintage variations added another layer to this narrative, with the year 2020 significantly shaping fungal communities through both temperature and precipitation, while 2021 exhibited a more tempered effect. These findings enhance our insights of the pathobiome dynamics, affirming that while it is evident that the weather of the actual year and season can influence microbial community dynamics, the mesoclimate of the particular *terroirs* plays a more significant role in shaping the overall diversity and composition of the microbial communities. These climatological variations were further depicted in Supplementary Figure S1 and Table 1 from Local Hungarian Meteorological Service (2023).

The uniformity of vineyard management practices, including consistent fungicide application, across all three *terroirs* during various seasons and vintages is a salient aspect of our study. While this uniformity minimizes direct impacts on the observed pathobiome variations, we acknowledge the potential indirect influence of fungicides on seasonal dynamics. Fungicide application introduces a dynamic element, influencing fungal community abundance and diversity, with documented short-term and long-term effects on non-target fungi (Leroux et al., 2002; Rodriguez-Morelos et al., 2021). Selective pressures on fungal populations, a known phenomenon in agricultural systems (Tranel and Wright, 2002), can result from fungicide applications, especially when timed with disease prevalence and seasonal factors. This aligns with findings in agricultural and environmental microbiology, highlighting fungicides’ capacity to shape the structure and diversity of microbial communities (Fournier et al., 2020; Zhang et al., 2020; Ma et al., 2021; Sun et al., 2023). While our study does not directly measure fungicide residues or their specific impact on fungal communities, recognizing fungicide application as a potential contributor to seasonal changes across *terroirs* suggests avenues for future research. Investigating the specific impacts and temporal dynamics of different fungicides on the pathobiome would enhance our understanding of factors shaping microbial communities in viticultural ecosystems.

Moreover, our network analysis suggested potential interactions, based on co-occurrence patterns, within these fungal communities. The unweighted analysis revealed differences in average degree, network density, and modularity across different parameters, pointing to the complexity and diversity of these communities. Bark samples exhibited higher average degree and modularity compared to soil and wood samples, possibly indicating a more complex network structure in this microhabitat due to its role as a critical overwintering site for various fungi and animals (Geiger et al., 2022).

Similarly, the weighted network analysis emphasized the role of the abundance data, revealing the importance of certain fungal genera such as *Diplodia* and *Eutypella*, as central ‘hubs’ in the network. These key players, often associated with GTDs, underscore the potential influence of disease dynamics on community assembly and vice versa.

To better understand these findings, it is crucial to interpret them in light of network metrics. The ‘degree’ of a node, representing the number of its connections, reflects its potential influence or importance within the network (Barabási and Oltvai, 2004). In our study, higher degrees in certain genera indicate their pivotal role in community interactions. Network density, which measures the proportion of actual connections relative to all possible connections, provides insights into the overall interconnectedness of the community (Newman, 2003). A higher density in our findings could imply a more integrated fungal community. Furthermore, ‘modularity’ assesses the degree to which the network can be partitioned into smaller, tightly connected groups or modules (Newman and Girvan, 2004). The higher modularity observed in bark samples suggests the existence of distinct microbial sub-communities or groups within that microhabitat. Biologically, more modular networks can contribute to system resilience, as disturbances might be confined within a single module, preventing them from affecting the entire system (Stouffer and Bascompte, 2011).

These observations support the notion that microbial interactions, which are effectively mapped through such network metrics, play a crucial role in the development and severity of grapevine diseases for this studied area. These interactions might involve synergistic or antagonistic relationships between co-infecting microbes (Hogan, 2006; Hosni et al., 2011; Lamichhane and Venturi, 2015).

Our findings offer valuable insights into the complex interplay between environmental factors and fungal communities in grapevines, supporting both our initial hypotheses. The metabarcoding, bioinformatics and network metrics suite of analyses conducted in this study enhances our understanding of the complex plant pathogenic fungal communities within grapevines and the environmental factors influencing them. Recognizing that the grapevine physiology and environment can strongly affect fungal behavior is crucial (Bruno and Sparapano, 2006; Freitas et al., 2009). By identifying relevant key players, understanding the possible effects of different parameters on fungal communities, and recognizing potential early indicators of disease, we are better equipped to devise effective strategies for managing impactful plant diseases in the fields. This knowledge can ultimately contribute to improved grapevine health and productivity. Building upon our research, there’s potential to not only advance the scientific understanding but also revolutionize grapevine cultivation and disease management strategies in the broader context of viticulture, creating a foundation for future studies, innovations and sustainable practices.

Looking forward, our study lays the groundwork for future investigations that could enhance our uncovering of the grapevine pathobiome. Firstly, a more in-depth exploration into the nuanced impact of vintage variations on fungal communities across different microhabitats would contribute significantly. Understanding how climatic conditions, especially during exceptional years, shape microbial interactions and disease dynamics could unravel critical insights. Additionally, delving into the direct impacts of fungicides, particularly their residues, on fungal communities could provide a more comprehensive understanding of their role in shaping seasonal variations. The network analysis opens avenues for studying microbial interactions further, emphasizing the need for experimental validations to decipher the nature of relationships indicated by network metrics. Investigating the specific responses of key genera, such as *Diplodia* and *Eutypella*, to environmental changes and their role in disease development could provide targeted strategies for disease management. Overall, this study serves as a steppingstone, and future research endeavors should capitalize on these insights to propel advancements in viticulture, offering innovative solutions for sustainable grapevine cultivation and disease control.

## Conclusion

This investigation brings to light the multifaceted dynamics of grapevine pathobiomes across varied microhabitats. It accentuates the substantial impact of both environmental and temporal determinants on these dynamics. Viewing this through the concept of “microbial *terroir*” lets us start to comprehend the critical role that spatial differences may play in molding the microbial environment surrounding grapevines. Our research, bolstered by advanced network-based approaches, which played a pivotal role in our analytical methodology, offers deeper insights into grapevine-microbe interactions for the specific area of study. This understanding is valuable, given its broader implications in wider agricultural and ecological spheres. Furthermore, our findings can serve as a guide to refine sustainable viticulture practices, with a potential focus on tailored plant health management. While this study stands as a milestone in demystifying the intricate relationship between grapevines and their resident microbes, specifically in Hungary, it also beckons toward a future rich with possibilities for even more detailed exploration in the realm of pathobiome interplay.

## Data availability statement

The datasets presented in this study can be found in online repositories. The names of the repository/repositories and accession number(s) are GenBank; accession numbers OQ370580-OQ371285.

## Author contributions

CL: Data curation, Formal analysis, Investigation, Methodology, Project administration, Resources, Software, Visualization, Writing – original draft, Writing – review & editing, Conceptualization, Validation. AG: Writing – review & editing, Data curation, Investigation, Validation. AM: Writing – review & editing, Investigation. KV: Resources, Writing – review & editing. GK: Writing – review & editing, Investigation. ZZ: Validation, Writing – review & editing, Investigation, Resources. JG: Conceptualization, Funding acquisition, Investigation, Methodology, Project administration, Supervision, Writing – review & editing, Resources, Validation.
