# Supplementary material for: Disentangling the effects of terroir, season, and vintage on the grapevine fungal pathobiome
Source: Front Microbiol. 2024 Jan 17;14:1322559. doi: 10.3389/fmicb.2023.1322559 (PMC10829339; doi:10.3389/fmicb.2023.1322559)
Supplement: Supplementary file 1 [file Data_Sheet_1.docx]

Supplementary Material

# Supplementary Tables

**Supplementary Table 1.** Co-occurrence derived from presence-absence matrices for fungal ASV pairs in different grapevine microhabitats. The probability values represent the likelihood of specific fungal pairs co-occurring frequently than expected by chance. Red color indicates a negative relationship, while blue signifies a positive relationship in the probability of co-occurrence column. There is the representation of species ASV code (sp1_name and sp2_name), species hypothesis (SH_1 and SH_2), genus and taxon attributed to the ASVs (sp1_genus, sp2_genus, sp. taxon_1, and sp taxon 2).

**Supplementary Table 2.** Comparative analysis of weighted network centrality metrics (Betweenness Centrality Range: 23.72 - 158.45, Degree Centrality Range: 2.17 - 4.42) in fungal pathogen communities across grapevine microhabitats, *terroirs*, seasons (Late Winter, Late Summer), vintages (2020, 2021), and health status.

**Supplementary Table 3.** Comparative analysis of unweighted network metrics (average degree range: 2.17 - 4.33, network density range: 0.018 - 0.072, modularity range: 0.202 - 0.457) in fungal pathogen communities across grapevine microhabitats, terroirs, seasons (late winter, late summer), vintages (2020, 2021), and health status.

**Supplementary Table 4.** Fungal ASVs considered as significant indicators of the different source types with their parameters, corresponding p-values, assigned functional guild, matching taxon, ITS2 rDNA sequence similarity (%) and taxonomic classification of the most similar matching sequence in the UNITE þ INSD dynamic Species Hypotheses database (version released in 2020). Only indicators with a known function are shown, displayed in the order of source type, parameters, and decreasing significance of indicator value.

## Supplementary Figures

**
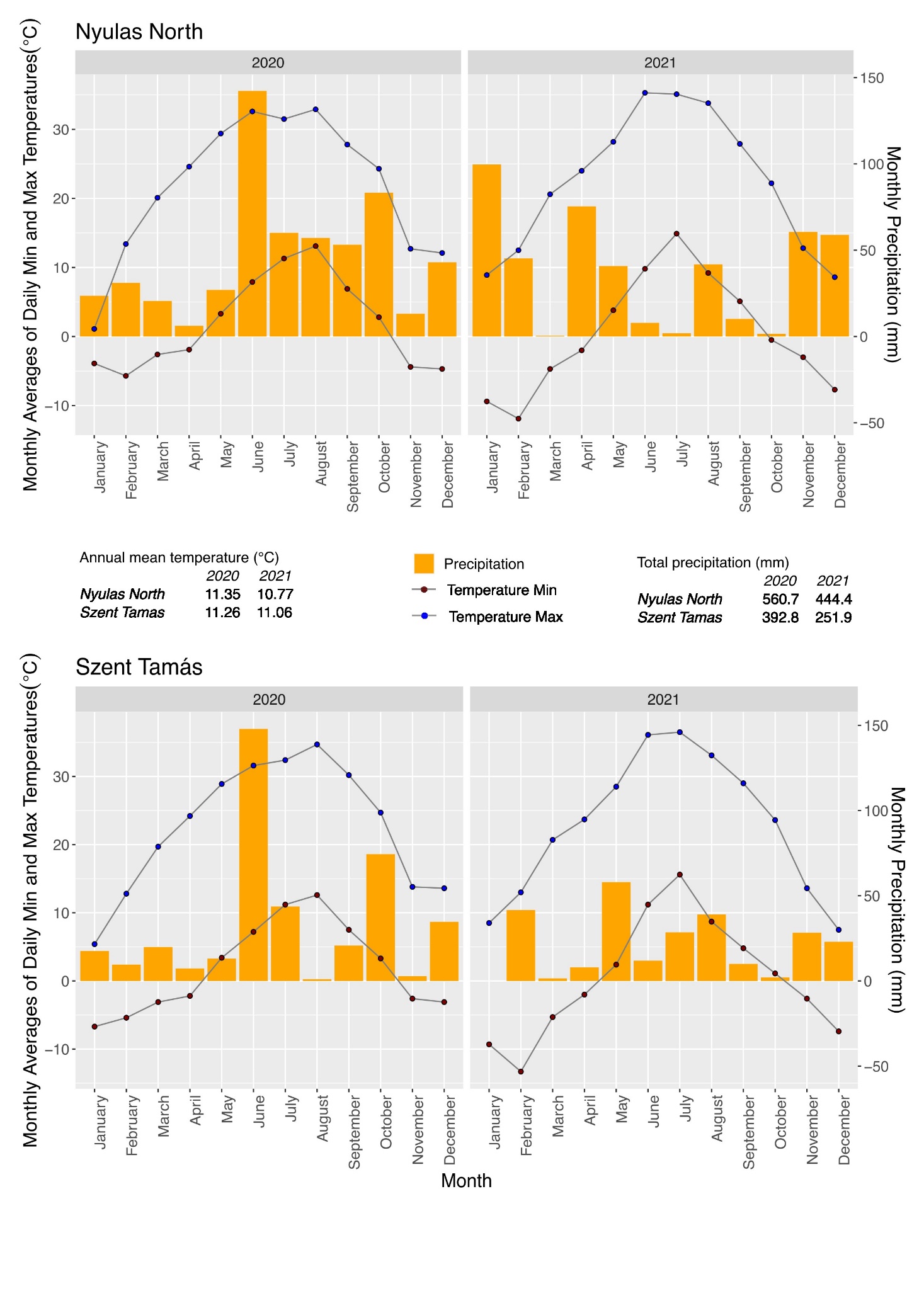
**

**Supplementary Figure 1.** Climatographical Data for Hungarian Regions (2020-2021). This chart represents the monthly averages of daily minimum and maximum temperatures (°C) and monthly precipitation totals (mm) for the regions of Nyulas North and Szent Tamas for the years 2020 and 2021. The annual mean temperature and total precipitation are also highlighted. Data were sourced from local meteorological stations, available at http://met.boreas2.hu/eke/, and visualized using the R package *'igraph'*.
